# Pharmacoequity in Anticoagulation Among Medicare Patients With Venous Thromboembolism

**DOI:** 10.1001/jamanetworkopen.2025.44529

**Published:** 2025-11-20

**Authors:** Amina A. Alkhalaf, Sungho Bea, Geetha S. Iyer, Julie C. Lauffenburger, Katsiaryna Bykov

**Affiliations:** 1 Division of Pharmacoepidemiology and Pharmacoeconomics, Brigham and Women’s Hospital, Boston, Massachusetts; 2 Department of Pharmacy Practice, College of Pharmacy, King Abdulaziz University, Jeddah, Saudi Arabia; 3 Harvard Medical School, Boston, Massachusetts; 4 Department of Population Medicine, Harvard Medical School and Harvard Pilgrim Health Care Institute, Boston, Massachusetts

## Abstract

This cohort study investigates the association of race and ethnicity with initiation of guideline-recommended direct oral anticoagulants in Medicare beneficiaries with venous thromboembolism.

## Introduction

Every year, more than 1 million US individuals experience venous thromboembolism (VTE),^1^ with higher VTE and mortality rates in Black patients suggesting disparities in care.^2^ While differences in uptake of direct oral anticoagulants (DOACs), which have replaced warfarin as the preferred treatment,^3^ have been observed in atrial fibrillation,^4,5^ little is known about patterns of DOAC adoption across racial and ethnic populations in VTE. We examined pharmacoequity, defined as equitable access to guideline-recommended medications regardless of race, ethnicity, gender, socioeconomic status, or geography, in DOAC use among Medicare beneficiaries with VTE.

## Methods

This cohort study was approved by the Mass General Brigham institutional review board and deemed exempt from consent because data were deidentified. This report follows the STROBE reporting guideline. We used Medicare fee-for-service claims (January 1, 2013, to December 31, 2022) to identify individuals aged 65 years or older who initiated an oral anticoagulant (warfarin, dabigatran, rivaroxaban, apixaban, or edoxaban) within 30 days of a VTE hospitalization and after at least 1 year of continuous Medicare Part A, B, and D coverage (eTable 1 in Supplement 1).

Exposure was Medicare-reported race and ethnicity obtained from the Social Security Administration. We analyzed Black, Hispanic, and White patients; individuals in other race categories (American Indian or Alaska Native and Asian or Pacific Islander) were excluded due to small numbers. The outcome was initiation of a DOAC vs warfarin. Multivariable logistic regression estimated odds ratios (ORs) for DOAC initiation in Black and Hispanic patients compared with White patients adjusting for 78 baseline covariates selected a priori for their association with anticoagulant prescribing and health care use (eTable 2 in Supplement 1). Demographics (age, sex, state of residence, and dual eligibility status) were captured at the index date. Analyses were conducted for the overall period and stratified into 2013 to 2015, 2016 to 2018, and 2019 to 2022. Cohorts were created using Aetion Evidence Platform version 4.53; analyses were conducted in June 2025 using SAS version 9.4 (SAS Institute).

## Results

Among 204 679 study-eligible individuals (22 139 Black [10.8%], 2942 Hispanic [1.4%], and 179 598 White [87.7%]; mean [SD] age, 77.3 [7.7] years; 124 292 female [60.7%]) (Table), 61.9% of White, 59.3% of Black, and 62.3% of Hispanic patients initiating anticoagulation were started on DOACs. Adjusted odds of DOAC initiation were lower in Black vs White patients overall (OR, 0.87; 95% CI, 0.84-0.90), in 2013 to 2015 (OR, 0.84; 95% CI, 0.80-0.89), and 2016 to 2018 (OR, 0.87; 95% CI, 0.83-0.93) but not in 2019 to 2022 or for Hispanic vs White patients throughout the study period (Figure).

**Table.** Selected Patient Characteristics

Characteristic	Patients, No. (%) (N = 204 679)			Absolute standardized difference vs White	
	Black or African American (n = 22 139)^a^	Hispanic (n = 2942)^a^	White (n = 179 598)^a^	Black or African American	Hispanic
Demographics					
Age, mean (SD), y	77.1 (8.0)	79.4 (8.6)	77.3 (7.7)	0.02	0.27
Gender					
Female	14 835 (67.0)	1985 (67.5)	107 472 (59.8)	0.15	0.16
Male	7304 (33.0)	957 (32.5)	72 126 (40.2)		
Medicare dual status					
Partial dual	2716 (12.3)	323 (11.0)	7680 (4.3)	0.29	0.26
Full dual	7727 (34.9)	2111 (71.8)	19 652 (10.9)	0.6	1.57
Index event					
Deep vein thrombosis	8485 (38.3)	1470 (50.0)	58 519 (32.6)	0.12	0.36
Pulmonary embolism	13 654 (61.7)	1472 (50.0)	121 079 (67.4)	0.12	0.34
Bleeding history and bleeding risk					
Any prior visit for bleeding	6736 (30.4)	919 (31.2)	53 873 (30.0)	0.01	0.03
HAS-BLED score, mean (SD)	2.6 (0.7)	2.5 (0.7)	2.4 (0.7)	0.38	0.26
Comorbidities					
Atrial fibrillation	3146 (14.2)	427 (14.5)	34 221 (19.1)	0.13	0.12
Congestive heart failure	8378 (37.8)	1030 (35.0)	51 556 (28.7)	0.2	0.14
Chronic kidney disease	7692 (34.7)	807 (27.4)	43 537 (24.2)	0.23	0.07
Diabetes	11 330 (51.2)	1634 (55.5)	58 731 (32.7)	0.38	0.47
Cancer	4856 (21.9)	515 (17.5)	39 895 (22.2)	0.01	0.12
Combined comorbidity score, mean (SD)	5 (3.4)	4.7 (3.4)	4.3 (3.2)	0.22	0.13
Frailty score, mean (SD)	0.2 (0.1)	0.3 (0.1)	0.2 (0.1)	0.25	0.37
Baseline medications					
Antiplatelets	2853 (12.9)	399 (13.6)	19 350 (10.8)	0.07	0.09
H2 antagonists	2840 (12.8)	465 (15.8)	16 430 (9.1)	0.12	0.2
Proton pump inhibitors	8562 (38.7)	1487 (50.5)	69 918 (38.9)	0.01	0.24
Nonsteroidal anti-inflammatory drugs	5577 (25.2)	969 (32.9)	38 791 (21.6)	0.09	0.26
Health care use: No. physician visits, mean (SD)	41.6 (35.8)	42.3 (34.0)	39 (31.1)	0.08	0.1

Abbreviation: HAS-BLED, hypertension, kidney or liver disease, stroke history, prior bleeding, unstable international normalized ratio, aged >65 years, and drug or alcohol use.

^a^ Race and ethnicity was available as a single variable in the data and obtained from the Social Security Administration.

**Figure.**
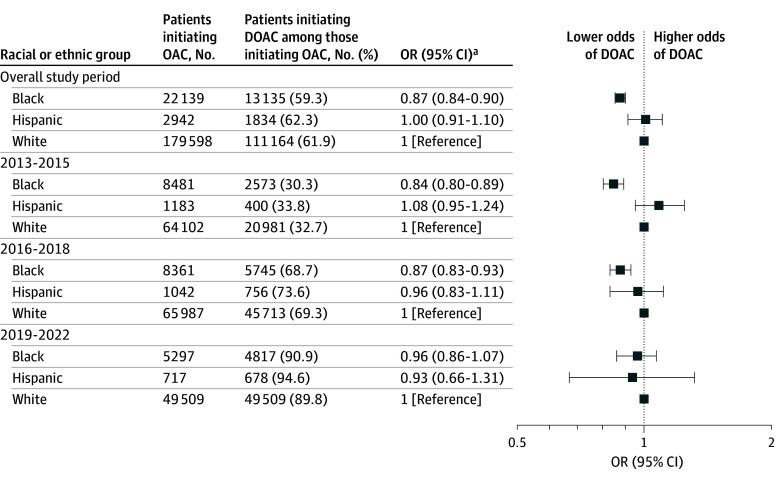
Odds of Direct Oral Anticoagulant (DOAC) Treatment by Race and Ethnicity Treatment with DOACs is shown across racial and ethnic groups in Medicare beneficiaries initiating oral anticoagulants after venous thromboembolism. OAC indicates oral anticoagulant; OR, odds ratio. ^a^Adjusted for all baseline covariates, including calendar year.

## Discussion

This cohort study found that Black patients had lower odds of being initiated on DOACs than White patients, especially during the early period of DOAC uptake. There was no difference in DOAC initiation odds between Hispanic and White patients. Our findings for Black patients are consistent with studies in atrial fibrillation^4,5^; however, DOAC adoption was similar in Hispanic and White patients in our study. Although absolute differences were small, even modest inequities can carry public health consequences given the high VTE incidence.

Our findings align with those of Nathan et al^6^ but expand generalizability by analyzing an older Medicare population in more recent years. In addition, we adjusted for a wide range of characteristics, ensuring comprehensive control for baseline differences.

Study limitations include the lack of information on patient or physician treatment preferences, prescriber-level factors, and other social factors influencing OAC choice, such as health literacy and cost. Medicare data combine race and ethnicity, and claims reflect dispensing rather than prescribing or use, which may result in misclassification. In addition, we did not assess disparities in anticoagulation initiation, including noninitiation due to nonadherence or a clinical decision not to treat. Moreover, findings may not be generalizable to other populations. Early adoption gaps found in this study suggest potential racial and ethnic disparities in access to newer pharmacotherapies.

## Notes

Supplement 1. **eTable 1.** Diagnosis codes used for VTE inclusion criteria **eTable 2.** List of study covariates

Supplement 2.
Data Sharing Statement
