# Supplementary material for: Pharmacoequity in Anticoagulation Among Medicare Patients With Venous Thromboembolism
Source: JAMA Netw Open. 2025 Nov 20;8(11):e2544529. doi: 10.1001/jamanetworkopen.2025.44529 (PMC12635871; doi:10.1001/jamanetworkopen.2025.44529)
Supplement: Supplement 1. — eTable 1. Diagnosis codes used for VTE inclusion criteria eTable 2. List of study covariates [file jamanetwopen-e2544529-s001.pdf]

Supplemental Online Content

Alkhalaf AA, Bea S, Iyer GS, Lauffenburger JC, Bykov K. Pharmacoequity in Anticoagulation among Medicare patients with venous thromboembolism. *JAMA Netw Open*. 2025;8(11):e2544529. doi:10.1001/jamanetworkopen.2025.44529

eTable 1. Diagnosis codes used for VTE inclusion criteria

eTable 2. List of study covariates

This supplemental material has been provided by the authors to give readers additional information about their work.

© 2025 Alkhalaf AA et al. *JAMA Network Open*.

eTable 1. Diagnosis codes used for VTE inclusion criteria

Inclusion	Hospital Diagnosis Codes	Care setting, Position
VTE (index VTE hospitalization)	**Deep vein thrombosis:**<br>ICD-9 DX: 451.1x, 451.2, 451.8x (except 451.82), 451.9, 452, 453.0, 453.1, 453.2, 453.4x, 453.6, 453.7x (except 453.71), 453.8x, 453.9<br>ICD-10 DX: I80.x, I81, I82.0, I82.1, I82.21x, I82.22x, I82.29x, I82.3, I82.40x, I82.41x, I82.42x, I82.43x. I82.44x, I82.45x, I82.46x, I82.49x, I82.4Yx, I82.4Zx, I82.50x, I82.51x, I82.53x, I82.54x, I82.55x, I82.56x, I82.59x, I82.5Yx, I82.5Zx, I82.60x, I82.62x, I82.70x, I82.72x, I82.A1x, I82.A2x, I82.B1x, I82.B2x, I82.C1x, I82.C2x, I82.89x, I82.9x	Inpatient, Primary
	**Pulmonary embolism:**<br>ICD-9 DX: 415.0, 415.1x<br>ICD-10 DX: I26.x	Inpatient, Primary

Abbreviations**:** VTE venous thromboembolism; ICD-9 International Classification of Diseases, 9th Revision; ICD-10 International Classification of Diseases, 10th Revision; DX diagnosis code.

© 2025 Alkhalaf AA et al. *JAMA Network Open*.

eTable 2. List of study covariates

Covariate	Definition
**Demographics**	
Age	Year of age at the time of dispensing, mean, standard deviation
Sex	Female, Male
Region	Northeast, South, Midwest, West
Medicare dual status	Partial dual, full dual
State	US state of residence
**Index**	
Index event	Deep vein thrombosis, pulmonary embolism, persistent provoked VTE, transient provoked VTE, unprovoked VTE
Calendar year	2015, 2016, 2017, 2018, 2019, 2020, 2021, 2022
**Bleeding history and bleeding risk**	
Any prior visits for bleeding	0, 1-2, >=3
HAS-BLED score	mean(sd)
Prior intracranial bleed	Yes, no
Prior gastrointestinal bleed	Yes, no
Prior other major bleed	Yes, no
**Lifestyle factors**	
Alcoholism	Yes, no
Obesity	Yes, no
Smoking	Yes, no
**Cardiovascular diseases**	
Arterial embolism	Yes, no
Atrial Fibrillation	Yes, no
Cardiac procedures	Yes, no
Congestive heart failure	Yes, no
Hyperlipidemia	Yes, no
Hypertension	Yes, no
Ischemic heart disease	Yes, no
Ischemic stroke	Yes, no
Cerebrovascular disease	Yes, no
Peripheral artery disease	Yes, no
Transient ischemic attack	Yes, no
**Other comorbidities**	
Chronic liver disease	Yes, no
Abnormal liver function	Yes, no
Anemia	Yes, no
Chronic kidney disease	Yes, no
Chronic kidney disease, stage ≥ 3	Yes, no
Coagulation defects	Yes, no
Diabetes	Yes, no
Peptic ulcer	Yes, no
Cancer	Yes, no
Combined comorbidity score	Mean(sd)
Frailty score	Mean(sd)
**Prior Medication**	
Antiplatelets	Yes, no
Angiotensin converting enzyme inhibitors	Yes, no

© 2025 Alkhalaf AA et al. *JAMA Network Open*.

Angiotensin receptor blockers	Yes, no
Antiarrhythmics	Yes, no
Antibiotics	Yes, no
Beta blockers	Yes, no
Calcium channel blockers	Yes, no
Diuretics	Yes, no
Digoxin	Yes, no
Influenza vaccine	Yes, no
H2 antagonists	Yes, no
Proton pump inhibitors	Yes, no
Other antacids: sucralfate	Yes, no
Nitrate vasodilators	Yes, no
Statins	Yes, no
Fibrates	Yes, no
Non-steroidal anti-inflammatory drugs	Yes, no
Estrogen/progestin	Yes, no
Selective serotonin reuptake inhibitors	Yes, no
Tricyclic antidepressants	Yes, no
Other antidepressants	Yes, no
Benzodiazepines	Yes, no
Insulin	Yes, no
Metformin	Yes, no
Sulfonylureas	Yes, no
Other oral hypoglycemic agents	Yes, no
**Healthcare Utilization**	
Number of Cardiologist visits	mean(sd)
Number of physician visits	mean(sd)
Number of emergency room visits	mean(sd)
Number of internal medicine visits	mean(sd)
Number of hospitalizations	mean (sd)
Bone mineral density test	Yes, no
Endoscopy	Yes, no
Home oxygen use	Yes, no
Mammography	Yes, no
Prostate specific antigen test	Yes, no
ECG & other cardiac imaging	Yes, no
Pap smear	Yes, no

The acronym HAS-BLED represents each of the bleeding risk factors and assigns 1 point for the presence of each of the following: hypertension (uncontrolled systolic blood pressure >160 mm Hg), abnormal renal and/or liver function, previous stroke, bleeding history or predisposition, labile international normalized ratios, elderly, and concomitant drugs and/or alcohol excess. The HAS-BLED scores range from 0 to 9, with scores of ≥3 indicating high risk of bleeding.

The HAS-BLED bleeding risk score in our claims-based study does not include labile INR, as laboratory data are not available in Medicare claims.
The Combined Comorbidity Score measures predicts short-and long-term mortality, by combining conditions in the Charlson and Elixhauser measures. The scores range from -2 to 26, with higher scores indicating high comorbidity burden.

Unless otherwise noted, characteristics were assessed during the baseline period, comprising the 365 days preceding initiation of an oral anticoagulant.

© 2025 Alkhalaf AA et al. *JAMA Network Open*.
