# Supplementary material for: Pharmacoequity in Anticoagulation Among Medicare Patients With Venous Thromboembolism
Source: JAMA Netw Open. 2025 Nov 20;8(11):e2544529. doi: 10.1001/jamanetworkopen.2025.44529 (PMC12635871; doi:10.1001/jamanetworkopen.2025.44529)
Supplement: Supplement 2. — Data Sharing Statement [file jamanetwopen-e2544529-s002.pdf]

Data Sharing Statement

Alkhalaf. Pharmacoequity in Anticoagulation Among Medicare Patients With Venous Thromboembolism. *JAMA Netw Open*. Published online November 20, 2025. doi:10.1001/jamanetworkopen.2025.44529

Data

Data available: Due to Data Use Agreement with Center for Medicaid and Medicare Services, patient-level data cannot be shared or made publicly available. Complete results data, study protocol, and programming codes are available from the corresponding author upon request.
